# The Ancient Neapolitan Sweet Lime and the Calabrian Lemoncetta Locrese Belong to the Same Citrus Species

**DOI:** 10.3390/molecules25010113

**Published:** 2019-12-27

**Authors:** Domenico Cautela, Maria Luisa Balestrieri, Sara Savini, Anna Sannino, Giovanna Ferrari, Luigi Servillo, Luigi De Masi, Annalisa Pastore, Domenico Castaldo

**Affiliations:** 1 Stazione Sperimentale per le Industrie delle Essenze e dei Derivati dagli Agrumi (SSEA), Azienda Speciale CCIAA di Reggio Calabria, via G. Tommasini 2, 89125 Reggio Calabria, Italy; dcastaldo@ssea.it; 2 Dipartimento di Medicina di Precisione, Università degli Studi della Campania “Luigi Vanvitelli”, Via L. De Crecchio 7, 80138 Napoli, Italy; marialuisa.balestrieri@unicampania.it (M.L.B.); luigi.servillo@unicampania.it (L.S.); 3 Stazione Sperimentale per le Industrie delle Conserve Alimentari (SSICA), Fondazione di Ricerca CCIAA di Parma, Viale Tanara 31/A, 43100 Parma, Italy; sara.savini.borsista@ssica.it (S.S.); anna.sannino@ssica.it (A.S.); 4 Dipartimento di Ingegneria Industriale e ProdALscarl, Università degli Studi di Salerno, Via Ponte Don Melillo 1, 84084 Fisciano, Italy; gferrari@unisa.it; 5 National Research Council (CNR), Institute of Biosciences and BioResources (IBBR), Via Università 133, 80055 Portici, Italy; luigi.demasi@cnr.it; 6 United Kingdom Dementia Research Institute Centre, Maurice Wohl Clinical Neuroscience Institute, Institute of Psychiatry, Psychology and Neuroscience, King’s College London, 125 Coldharbour Lane, London SE5 9NU, UK; annalisa.pastore@crick.ac.uk; 7 Ministero dello Sviluppo Economico (MiSE), Via Molise 2, 00187 Roma, Italy

**Keywords:** agrobiodiversity, lemoncetta Locrese, Mediterranean sweet lime, Neapolitan limmo, Neapolitan “four citrus fruits” liqueur, taxonomy

## Abstract

“Neapolitan limmo” is an ancient and rare sweet Mediterranean lime, now almost extinct but used until a few decades ago for the production of a fragrant liqueur called the “four citrus fruits”. The objective of this work was to compare, through the use of chemical (flavonoids, volatile organic compounds, and chiral compounds) and molecular (DNA fingerprint based on RAPD-PCR) markers, the residual population of Neapolitan limmo with other populations of sweet limes, identified in Calabria and known as “lemoncetta Locrese”. We report for the first time specific botanical characteristics of the two fruits and unequivocally show that the ancient sweet Mediterranean limes Neapolitan limmo and lemoncetta Locrese are synonyms of the same Citrus species. Owing to the biodiversity conserved in their places of origin, it will now be possible to recover, enhance and implement the use of this ancient sweet lime for agro-industrial purposes.

## 1. Introduction

**Figure 1:**
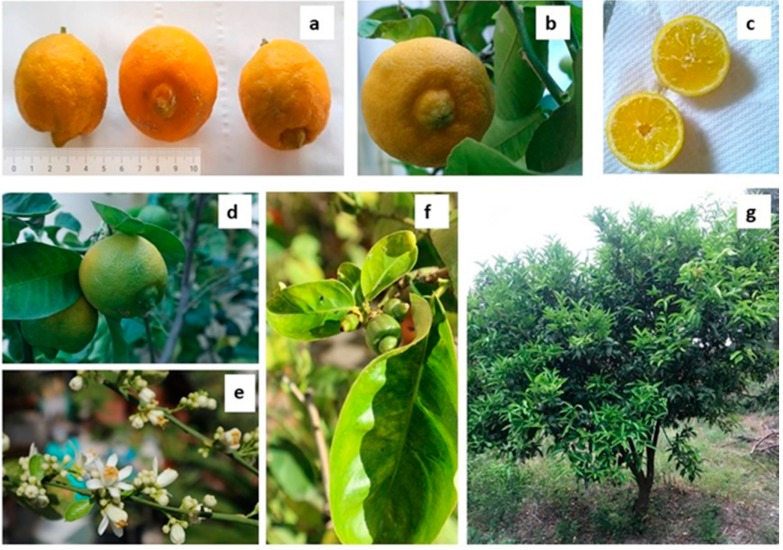
Neapolitan limmo or lemoncetta Locrese. (**a,b**) Ripe fruits are sulfur-yellow in color and have a diameter of 4–5 cm. (**c**) Fruit section with 8–10 loggias. (**d**) Ripe fruits with greenish color. (**e**) Young leaves and flowers. (**f**) Leaves and small fruits. (**g**) Neapolitan limmo tree.

“Limmo” or “limo” or also “limma”, not to be confused with the more famous lime (*C. aurantifolia*), is an ancient Neapolitan citrus of the genus *Citrus*. The first traces of the presence of limmo in the Neapolitan province date back to the end of the seventeenth century [1]. It was described as the fruit of Lomia or Lumia, a species of sweet and sweet-smelling *Citrus* fruits similar to lemon but smaller [2]. Limmo has a strongly rounded shape of about 5–6 cm diameter (Figure 1a). According to TG/203/1 UPOV guidelines [3], it is morphologically characterized by a base with a depressed, slightly rounded distal part and a nipple of conical and umbonate shape, sunken at the base of ca. 1–2 cm (Figure 1b). It is similar to the “Sicilian lumia”, recently described by Raimondo et al. [4].

Limmo has a thin yellow skin (Figure 1c) in the ripe fruit, consisting of 8–10 loggias containing few seeds, with segments of color between yellow and green, of delicate flavor, not sour, aromatic and sometimes also with greenish notes in the peel of the ripe fruit (Figure 1d). The limmo flowers, compared to lemon, are on average smaller, of medium size, fragrant, with white petals and buds (Figure 1e,f). Small is also the limmo tree with leaves similar to those of lemon (Figure 1f,g). The cultivation of this sweet lime is now completely amateurish. We could count only a few plants in gardens of Naples and of the Neapolitan province.

Limmo belongs to the group of the Mediterranean sweet limes and lemons [5]. It appears nevertheless distinct from them for the color of the flower petals and for the low acidity of the fruit juice [4,6,7]. The acidity is instead high in lemons (*C. limon*) and medium high in most of the common limes (*C. limetta* Risso, subsect. *Limonoides*) [8], like the acidic “limonette de Marrakech” [9], and the Mediterranean sweet lime, *C. lumia* Risso. The latter is an acid-less variety of *C. limetta* Risso, (subsect. *Decumanoides*-sect. *Citrophorum* according to Tanaka) [8] which has a long history of cultivation in Italy as early as the seventeenth and eighteenth centuries [10,11]. The acid-less phenotype of *C. limetta* is due to the inability of producing anthocyanin pigments in leaves and flowers and proanthocyanidins in seeds [6,7] with low citric acid contents [12,13,14] and juice pH values also above 6 [5,6,7,15].

Neapolitan limmo stands out among other citrus fruits for its fragrant aroma. The flavor is not very sweet, rather watered down, almost totally devoid of acidity, not savory and, therefore, unappetizing; the latter characteristics were in ancient times systematically exploited in Naples and its surroundings as a defense from thieves. At the end of the nineteenth century, traditional and patrician gardens were surrounded by limmo trees to protect the property from the street urchins, who were Neapolitan boys accustomed by the adversity and poverty of that time to survive in the street thanks to small daily thefts like stealing seasonal fruits from city gardens. Limmo was also used in Neapolitan families to prepare the ancient liqueur “with the four citrus fruits”, today almost disappeared. The liqueur was obtained by cold maceration in ethylic alcohol of the slightly unripe peels of oranges, mandarins, lemons, and Neapolitan limmo. This ancient liqueur was much more sought after than the more popular and widespread liqueur “limoncello”, another Neapolitan liqueur obtained using IGP lemons (Protected Geographical Indication) from the Amalfi coast and Sorrento [16,17].

The use of limmo in natural medicine, together with that of other citrus fruits, was reported in the 1825 edition of Phamacopoeia by Antonio Ferrarini (pharmacist, Member of the Health Commission of Bologna City and surroundings, and Lecturer at the Faculty of Pharmacy) [18]. Along with other citrus fruits, limmo was used for the preparation of “aromatic cedar water” or “citron aromatic water”. In traditional medicine, the limmo juice was once used as a remedy for cough mixed with prickly pear juice [19].

No studies on limmo are present in the literature. Early taxonomists hypothesized that lemons and limes are derivatives or hybrids of citrons. However, a definitive classification and origin of the species was not proposed. It is a shared opinion that cultivated limes, sweet limes, and lemons originate from interspecific hybridization of cedar (*C. medica* L.) in combination with sour orange (*C. aurantium* L.) while the *C. maxima* × *C. reticulata* hybrid gives rise to the *C. limettioide* subgroup Palestinian sweet lime and *C. meyeri* Meyer lemon [5,20].

Since in Naples limmo was also described as a “sweet bergamot”, we searched for this sweet lime in Calabria as well, with the intention of verifying the presence of limmo. Calabria is a region of Southern Italy with extensive citrus fruit cultivations, especially in the Ionic area of the province of Reggio Calabria, the area of origin and production of bergamot (*Citrus Bergamia* Risso) [21,22].

The results of this survey showed the presence of a discreet population of sweet limes in the region East to Reggio Calabria, the Locri area. The local fruit is morphologically similar to Neapolitan limmo and locally known as “lemoncetta Locrese” or “pirettu Locrese”. This fruit is of no agro-industrial use and thus of little economic importance. As for Neapolitan limmo, it was never characterized compositionally.

We thus decided to compare the populations of Neapolitan limmo with the Calabrian lemoncetta Locrese with chemical and genetic approaches. We measured chemical markers of citrus fruits, such as glycoside flavanones and determined the profile of volatile organic compounds (VOCs) in juice and peel and the enantiomeric distribution of volatile organic chiral compounds. We then analyzed the genetic diversity of the two populations by Random Amplified Polymorphic DNA (RAPD) analysis.

The results of this study aimed at characterizing and comparing the two apparently distinct fruits led us to conclude that they are compositionally and genetically indistinguishable within variations due to climatic and soil differences. These results will help to restore the use of the ancient Mediterranean limmo to produce the Neapolitan “four citrus fruits” liqueur and as a promising and precious resource for the essential oil industry.

## 2. Results and Discussion

### 2.1. Flavonoids, Organic Acids and Proximate Constituents

Flavanones and to a lesser extent flavonols are the predominant flavonoids in the genus *Citrus* [23]. The quali-quantitative distribution of these phenols is largely influenced by the specie and/or the variety [24,25,26]. Flavonoids are thus commonly used as chemotaxonomic markers and evaluate the quality and genuineness of citrus juices [24,27,28,29,30]. Since flavanones constitute virtually all of the total flavonoids present (e.g., 98% in grapefruit, 90% in lemons, and 96% in limes) [29], we focused on the major aglycone flavanones with their rutinose or neohesperidose glycosides as markers to differentiate Neapolitan limmo and lemoncetta Locrese from other citrus juices and between the two populations. The same approach was utilized by Mouly et al. [24] to effectively differentiate lemon and lime, varieties of grapefruits (white, pink, red, and green), and sweet oranges (Valencia, navel, blood, Thomson, and Malta). The flavonoid profile is also a method widely utilized to detect the possible mixture of different juices as for instance the addition of bergamot to lemon juice [30]. The range of variability of flavonoids and organic acids for citrus juices are reported in the code of practice of the International Federation of Fruit Juice Producers (IFFJP).

**Figure 2:**
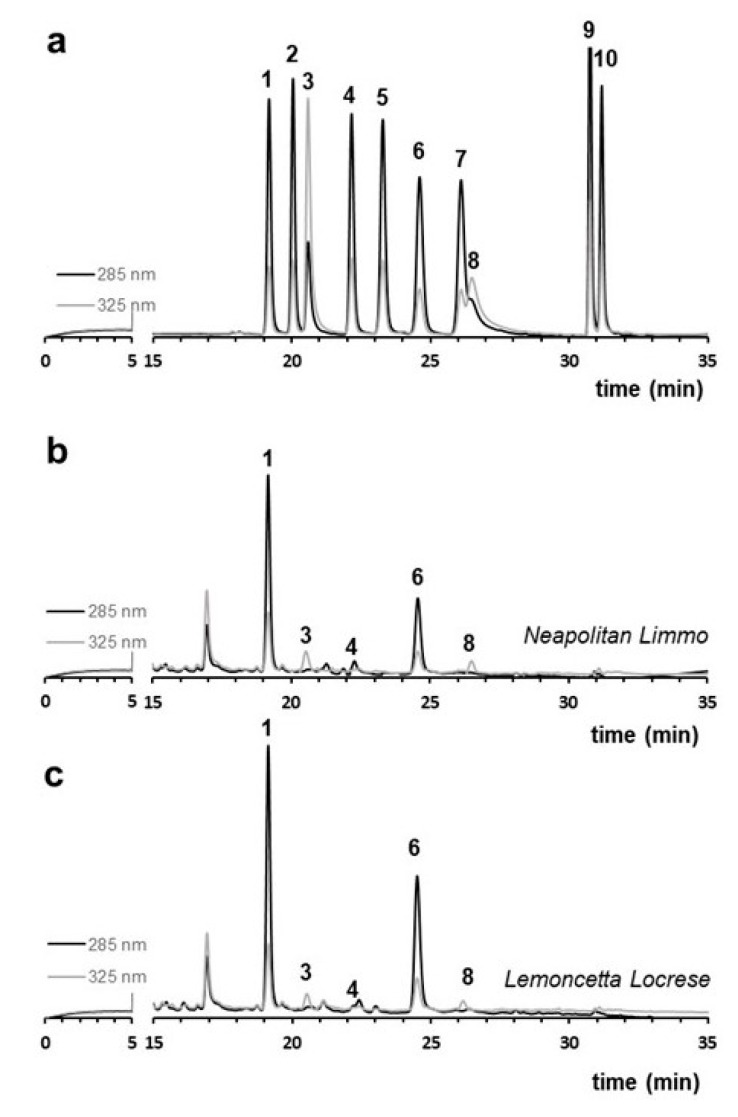
Liquid chromatography (LC) flavonoid profile of Neapolitan limmo and lemoncetta locrese. (**a**) Standard containing a mixture (50 mg/L) of eriocitrin (1), neo-eriocitrin (2), rutin (3), narirutin (4), naringin (5), hesperidin (6), neohesperidin (7), diosmin (8), poncirin (9), didymin (10). (**b**) LC chromatograms of Neapolitan limmo (**c**) LC chromatograms of lemoncetta Locrese. Flavanones were monitored at 285 nm (black line), flavones at 325 nm (gray line).

We thus compared by HPLC the population of flavonoids in Neapolitan limmo with those of lemoncetta Locrese (Figure 2 and Table 1). An identical flavonoid profile unites both analyzed populations (Figure 2). Neapolitan limmo had flavanone profiles more like lemoncetta Locrese. The insignificant difference (*p* < 0.05) observed in flavonoid contents are within the normal limits of environmental variability of these two juices.

Both Neapolitan limmo and lemoncetta Locrese are qualitatively characterized by a common presence of five different flavonoids: the three rutinosidic flavanones, hesperidin, eriocitrin, and narirutin and the two flavones *O*-glycosides, rutin and diosmin. The identification of these flavonoids is confirmed not only by the retention times but also by spectra analysis compared to their standards (data not shown). The samples do not contain flavanone *O*-neohesperidose and the non-bitter flavanone neoponcirin.

Of particular interest between the present flavonoids is eriocitrin, a flavonoid that is exclusively characteristic of lemon juice [27,29], and is almost absent in orange and grapefruit juice. Eriocitrin in limmo and lemoncetta strengthens the genetic closeness to lemon of these Mediterranean limes, both hybrids of citron (*C. medica* L.) being a sour orange × Citron cross [5].

The flavonoid composition of limmo and lemoncetta was also compared with that of two other limes, both previously characterized by Nogata et al. [23]. The first is classified under the *Limonoides* subset according to the Tanaka’s system [8], with the common name Sweet lemon and the scientific name *C. limetta* or *C. limetta* Risso, with slightly acidulous pulp [9,12,13]. The second is classified in the subsection of *Decumanoides* (sect. *Citrophorum*) [8], with common name lumie and the scientific name *C. lumia* or *C. lumia* Risso, with sweet and non-acidic pulp [4,13]. This citrus is most diffused in Italy and in some southern regions of France [31]. A prevalent similarity emerges from the comparison between the flavonoid profiles of *C. limetta* and *C. lumia* and those of Neapolitan limmo or lemoncetta Locrese (Table 1): Neapolitan limmo, lemoncetta Locrese and *C. lumia* have a significant content of the flavanone *O*-rutinosides esperidine and eriocitrin. They also have a reduced content of the other flavanone rutinosides narirutin (Table 1) and of the flavones rutin and diosmin. Significative is the common absence of the flavanones neoponcirin, naringin, neohesperidin, neoeriocitrin e poncirin in limmo, lemoncetta and *C. lumia* but not in *C. limetta*.

Unfortunately, no other paper on the traditional Italian sweet lime varieties besides Nogata et al. [23] report data on flavonoids [12,13]. The data are anyway consistent with those recently found by Smeriglio et al. [32] on *C. Lumia* which reported a similar significant presence of hesperidin and eriocitrin.

A further indication that the Neapolitan limmo can be with good reasons classified in the *Citrus lumie* group is also offered by two recent papers by two distinct research groups [6,7]. These authors demonstrated by independent methodological approaches that in acid-less varieties of citrus, exceptionally low fruit acidity is associated with absence of anthocyanin pigments in leaves and flowers and of proanthocyanidins in seeds and flowers without pigmentation or white, like those of Neapolitan limmo (Figure 1e).

**Figure 3:**
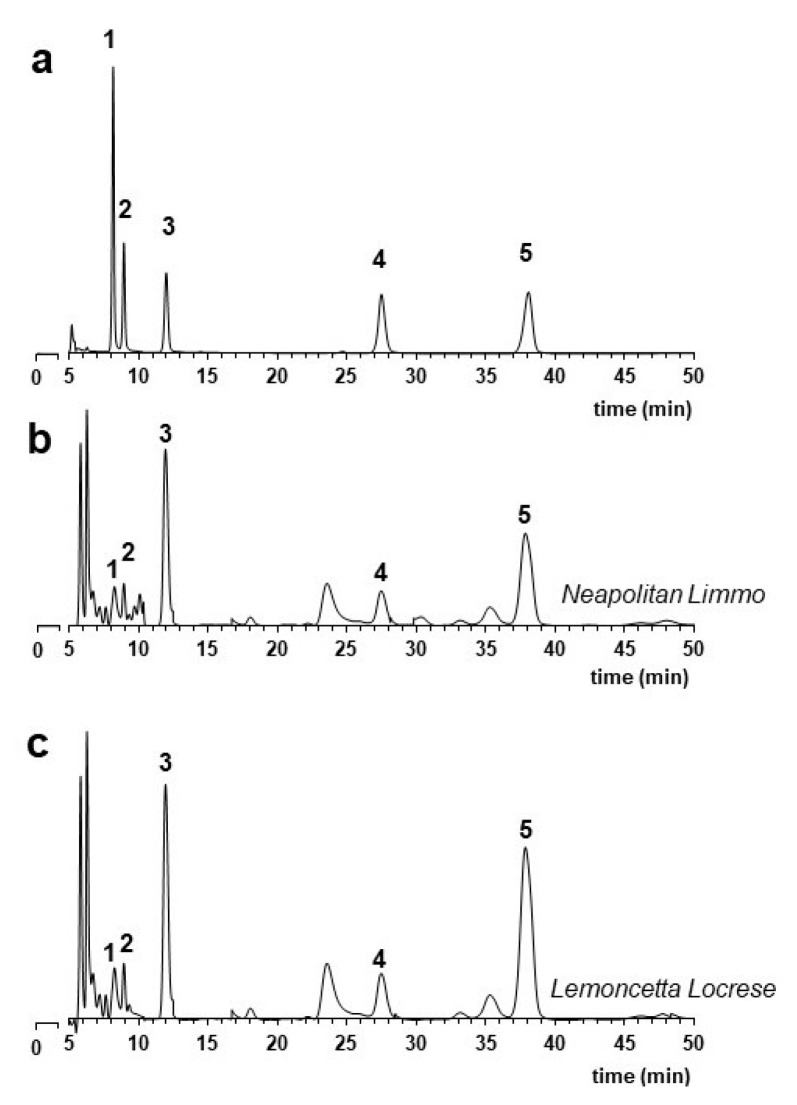
Organic acid profile of Neapolitan limmo and lemoncetta locrese. (**a**) Standard containing a mixture of tartaric acid (1) 0.5 mg/mL; quinic acid (2) 0.5 g/L; malic acid (3) 0.5 g/L; citric acid (4) 0.5 g/L; fumaric acid (5) 2.5 mg/L. (**b**) LC chromatograms of Neapolitan limmo (**c**) LC chromatograms of lemoncetta Locrese.

Next, we extended our investigation to the quali-quantitative distribution of organic acids, the overall acidity, and the pH of the juice. These parameters can give useful indications on the nature of the lime type discriminating between acid ecotypes. Both limmo and lemoncetta have qualitatively a common acidic chromatographic profile characterized by the presence of five organic acids: malic, citric, quinic, tartaric, and fumaric acids (Figure 3). Similar are also the quantitative data (Table 2).

Malic acid is the dominant organic acid of these sweet Mediterranean limes with average values of 1.57 ± 0.03 g/L in Neapolitan limmo and slightly higher, 1.88 ± 0.02 g/L, in lemoncetta. It is probably this significant presence in the acidic profile that confers to the juices of these fruits (acid-less sweet tasting) that smooth tartness acidity given by malic acid. This taste is clearly different from the sensorial sour quality given by citric acid in juices when this is dominant [33].

Both Neapolitan limmo and lemoncetta showed reduced contents of citric acid with average values of about 0.94 g/L in the group of Neapolitan limmo and even lower in lemoncetta Locrese (0.48 g/L), with an average pH > 5.7 and values of total acidity < 1.4 g/L (Table 2). This is consistent with the phenotypes of the sweet forms of *C. limetta* Risso—Mediterranean sweet lime—sweet Roman [12], Roman [13,34], Lima Dulce, or Dulce lime [14].

Quinic acid is the most expressed acidic compound after malic acid and citric acids. The average levels of malic acid are between 0.10 and 0.43 g/L with higher average values for lemoncetta Locrese compared to Neapolitan limmo (Table 2). Also, for this acid, the quantitatively expressed levels appear on average higher in the group of lemoncetta Locrese than in those of Neapolitan limmo. The average values are however completely comparable with each other. Finally, fumaric acid is much less expressed and generally does not exceed 0.01 g/L.

This analysis makes us conclude that limmo and lemoncetta are chemically similar although there is an appreciable quantitative difference in some substances likely due to climatic and soil composition and other environmental differences. The values of flavonoids and other metabolites are, for instance, different likely because of the different degree of activivity of phenylalanine ammonium lyase, the enzyme central to the production of the biosyntesis precursor of flavonoids cinnamic acid [35].

### 2.2. Chirospecific Analysis

**Figure 4:**
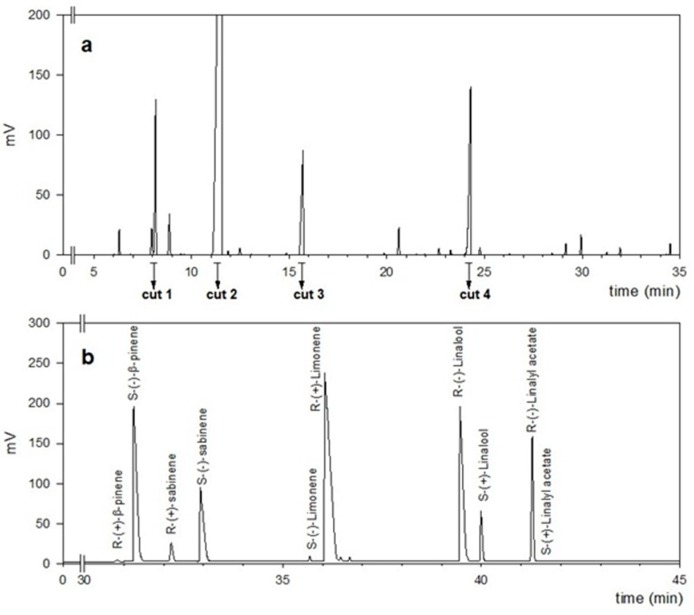
Analysis of the chirality of Neapolitan limmo and lemoncetta Locrese (**a**) GC chromatogram of Neapolitan limmo essential oil with the four heart-cuts. (**b**) Enantio-MDGC chromatogram of selected chiral compounds in Neapolitan limmo.

Biological activity is often correlated with chiral properties. In citrus fruits, chiral compounds are widely used as indicators of adulteration or fraud of essential oils by addition of synthetic or natural compounds of different botanical origin. The GC profiles of volatile aromatic compounds of essential oils from Neapolitan limmo and lemoncetta Locrese (Figure 4a) were initially compared and analyzed by heart-cutting multidimensional GC [36] to estimate the enantiomeric distribution (ee%) of chiral β-pinene, sabinene, limonene, linalool and linalyl acetate (Figure 4b).

As for flavonoids and organic acids, an identical metabolic profile of volatile compounds was common to both citrus populations (Figure 4a). Forty-three volatile aromatic compounds were identified. In both populations, the more expressed were limonene (61.8 ≥ ± 14.4%) ≥ linalyl acetate (9.2 ± 0.5%) ≥ linalool (6.6 ± 0.4%) ≥ β-pinene (4.4 ± 2.9%) ≥ myrcene (1.3 ± 0.6%) ≥ sabinene (0.8 ± 0.4%) ≥ α-terpineol (0.7 ± 0.6%) ≥ α-pinene (0.5 ± 0.4%) ≥ geranial (0.4 ± 0.3%) ≥ neral (0.3 ± 0.1%) ≥ β-bisabolene (0.2 ± 0.1%) ≥ nerol (0.2 ± 0.1%) ≥ terpinene and citronellol ranged from 0.05 to 0.1%. Camphene, octanal, α-phellandrene, terpinolene, and terpinen-4-ol were less than 0.05%. 

The data obtained by four heart-cut multidimensional GC are even more interesting; the enantiomers of β-pinene, sabinene, limonene, linalool, and linalyl acetate were all well-separated on a DiActButylsilyl γ-CDX chiral column (Figure 4b). The dominant enantiomeric form for limonene was (*R*)-(+). Both populations showed (*R*)-(+) for β-pinene and sabinene (*S*)-(−), and (*R*)-(−) for linalyl acetate and linalool (Table 3).

### 2.3. Volatile Organic Compounds Analysis

**Figure 5:**
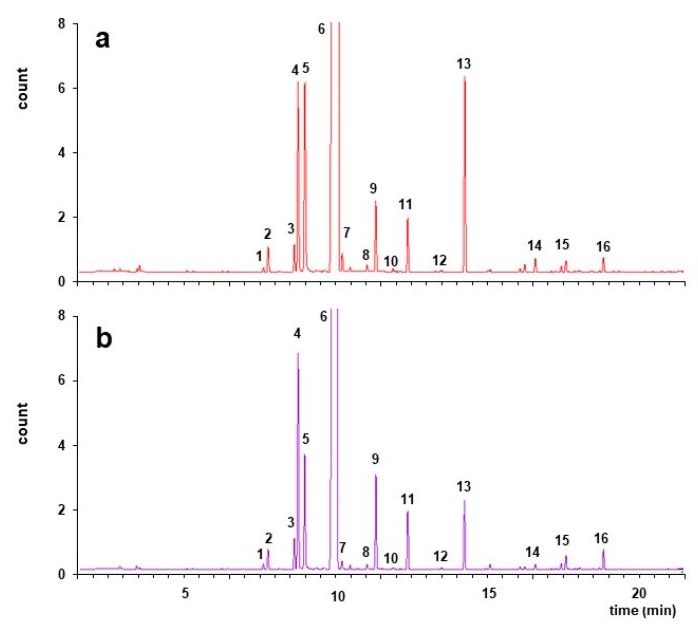
Total ion chromatograms of the juices collected by SPME/GC/MS. (**a**) Neapolitan limmo. (**b**) lemoncetta Locrese. IS: Internal Standard. The peak numbers correspond to the numbers in the first column of Table 4.

**Figure 6:**
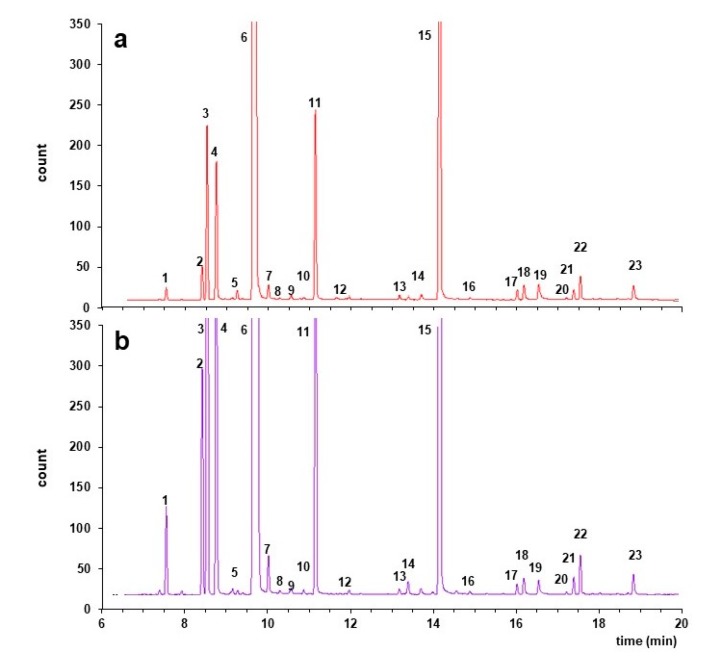
Total ion chromatograms of the peels collected by SPME/GC/MS. (**a**) Neapolitan limmo. (**b**) lemoncetta Locrese. Peak numbers correspond to the numbers in the first column of Table 5.

Comparison of the total ion chromatograms of the aroma components collected in the juices of Neapolitan limmo and lemoncetta Locrese showed the presence of 8 terpenes, 5 monoterpenoid alcohols, and 3 sesquiterpene hydrocarbons in the juices. Both fruits presented the same volatile compounds (Figure 5 and Figure 6).

In the variety lemoncetta Locrese 12 volatile compounds were in concentrations significantly higher than in limmo (Table 4).

D-limonene was the main flavor compound found in both varieties, but its concentration was significantly higher in Neapolitan limmo compared to lemoncetta Locrese (*p* < 0.05). Other major components (except limonene) are β-pinene, β-myrcene, and bergamol (linalyl acetate) for both varieties. The last two compounds are significantly higher in Neapolitan limmo (*p* < 0.05). Moreover, other volatile aromas found at small percentage such as α-phellandrene, α-pinene, β-phellandrene, linalool, trans-α-bergamotene, and β-bisabolene were considered to be important compounds influencing the entire aroma [37]. Their concentrations were significantly higher in lemoncetta (*p* < 0.05). Only the volatile compound α-pinene was not significantly different in the two varieties (*p* > 0.05).

Twenty-three volatile compounds were identified in the peels: 10 terpenes, 8 monoterpenoid alcohols, and 5 sesquiterpene hydrocarbons (Table 5). Both varieties exhibited the same volatile compounds with different intensity (Figure 6). The peel of lemoncetta Locrese presented significantly higher areas than Neapolitan limmo. The main component of the peels is Limonene followed by bergamol, linalool, β-pinene, and β-myrcene with different intensities. Limonene value was not reported because is no longer linear and the areas were off the charts. Other relevant compounds are α-pinene, β-phellandrene, β-pinene, β-myrcene, linalool, and bergamol which are significantly higher in lemoncetta compared to Neapolitan limmo (*p* < 0.05). Only two volatile compounds (nerol acetate and geraniol acetate) are significantly higher in Neapolitan limmo than in lemoncetta Locrese (*p* < 0.05).

The compounds found in these varieties were also reported in other Citrus varieties. Particularly, in the peels were found 10 additional volatile compounds: cis-β-terpineol, terpinolene, α-terpineol, acetic acid octyl ester, trans-geraniol, α-terpineol acetate, nerol acetate, geraniol acetate, α-bergamotene, and cis-α-bisabolene. The absence of these compounds in the juices is probably due to the juice squeezing. The extraction pressure conditions will determine different aroma components in juices and in peels.

Our results revealed that there are not qualitative differences between the two varieties. The aromatic profiles are identical and there are not specific volatile compounds that could be used to differentiate the varieties. The main differences are connected only to the intensity of the aromatic profile. The use of SPME-GC-MS thus resulted to be a valuable tool to analyze the volatile profile of the two sweet lime juices and peels and obtain a quality characterization of fruits from different varieties.

### 2.4. Genetic Comparison by DNA-Based Molecular Markers

The genetic similarity of the two Mediterranean sweet lime populations was finally analyzed using RAPD molecular markers [38]. This technique was preferred to DNA barcoding and phylogenic analysis because DNA barcoding works best if the sequences have sufficiently diverged. We feared that the assay could prove inconclusive in our case given the indications from chemical data that the evolutionary distance between Limmo and lemoncetta is close [39]. RAPD has instead proven useful in the identification of Citrus cultivars and the assessment of genetic relatedness for neglected or little-known citrus accessions [40,41]. Lemon (*C. limon*) cultivars of Campania (Italy) were for instance distinguished by their RAPD profiles using five arbitrary primers, confirming that RAPD markers can successfully identify lemon genotypes [42]. Iannelli et al. [43] characterized lemons by combining genome size and RAPD markers. In their work, the primer U19 utilized for distinguishing lemon genotypes.

**Figure 7:**
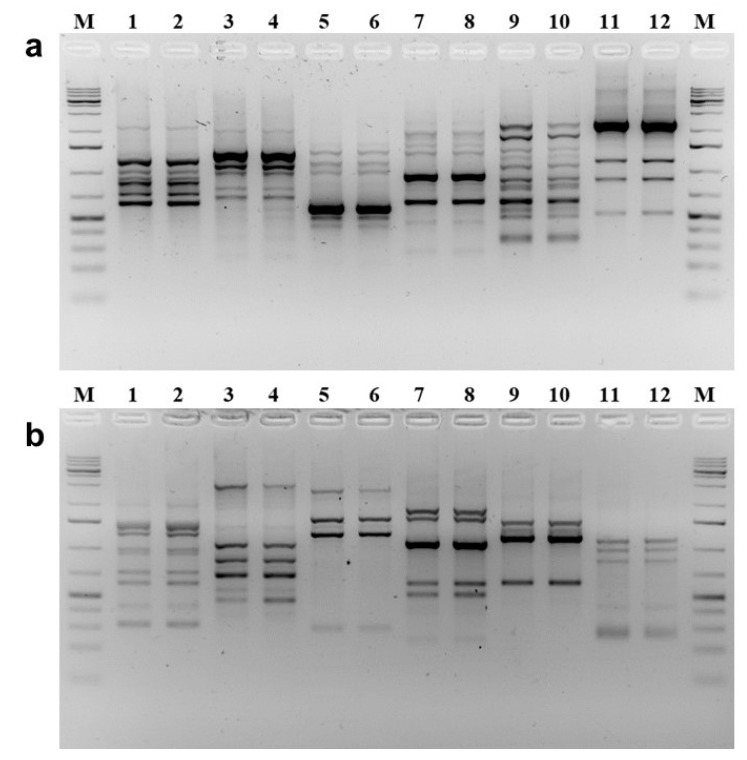
Genetic diversity of Neapolitan limmo and lemoncetta Locrese by RAPD molecular analysis. Comparison of the RAPD results on agarose gels of lemoncetta Locrese (odd lanes) and Neapolitan limmo genomes (even lanes), respectively, with the indicated arbitrary primers. (**a**) Lanes 1, 2: primer A05; lanes 3, 4: primer AK10; lanes 5, 6: primer AN10; lanes 7, 8: primer AX01; lanes 9, 10: primer AX08; lanes 11, 12: primer G07. (**b**) Lanes 1, 2: primer G12; lanes 3, 4: primer G19; lanes 5, 6: primer E10; lanes 7, 8: primer E11; lanes 9, 10: primer U4; lanes 11, 12: primer U19. Lanes M: GeneRuler 1 kb Plus DNA Ladder (Thermo Fisher Scientific) as molecular weight marker, containing three darkest bands consisting of 5000, 1500, and 500 bp.

The RAPD-PCR method allowed the genetic analysis of Neapolitan limmo and lemoncetta Locrese by simply comparing the presence/absence of bands in DNA amplification patterns, visible after electrophoresis on agarose gel, as the bands represent the numerous loci detected randomly and dispersed in their respective genomes. We used primers already successfully considered for the variety discrimination of other species [44,45] but we obtained more markers as compared to previous work. The primers used showed high reproducibility of amplification products. They also showed the absence of polymorphisms in DNA in the comparison between limmo and lemoncetta and therefore the impossibility to discriminate between the two populations (Figure 7a,b).

The RAPD profiles obtained with each primer were extremely different from each other, allowing us to explore different regions of the genome. Alleles corresponding to reproducible amplicons, given the dominant genetic nature of RAPD markers, identified a total of 80 markers or loci (Table 6). The number detected was dependent on the primer used but not on the variety, with an average of 6.7 loci per primer, going from the minimum of four bands for primers G07 and U4 and at most 12 bands for the AX08 primer. Also, the genetic analysis suggested that the Neapolitan limmo and the lemoncetta Locrese are likely synonyms of the same variety since the loci were not polymorphic and did not allow to discriminate between the two local varieties.

## 3. Materials and Methods

### 3.1. Plant Materials

Fruits and Leaves samples of Neapolitan limmo and lemoncetta Locrese were harvested in January 2018 and 2019 from populations located in areas of Afragola (40°55′37′′ N; 14°18′42′′ E), Pozzuoli (40°49′39′′ N; 14°9′11′′ E) and Pianura (41°02′24′′ N; 14°11′09′′ E) (Campania, Italy) or Locri (38°14′19′′ N; 16°15′34′′ E) and Siderno (38°16′ N; 16°18′ E), Reggio Calabria (Calabria, Italy), respectively, and placed in a 4 °C refrigerated box to be processed.

Chemicals: All chemicals were purchased from Sigma-Aldrich (St. Louis, MO, USA) or Extrasynthes (Genay, France). An internal standard solution of camphor used for the analysis of volatile compounds in the juices was obtained from Sigma-Aldrich. The purity of all of the standards was beyond 95%. All other solvents and reagents were of analytical grade.

### 3.2. Preparation of the Samples for Chemical Analyses

Chemical analyses were performed on juices obtained in the laboratory from fresh fruits of Neapolitan limmo and lemoncetta Locrese. A total of 6 different Neapolitan limmo juice samples were used (2 in 2018 and 4 in 2019) and 8 lemoncetta Locrese samples (3 in 2018 and 5 in 2019). The juices were prepared using a manual squeezer, filtered through a stainless-steel filter with 1.18 mm mesh diameter, centrifuged at 18,000× *g* for 60 min at 4 °C, placed in plastic bags in 100 mL aliquots, and stored at −20° C until usage. The essential oils were extracted from fruits of limmo or lemoncetta (2 kg) through manual abrasion of the frozen flavedo at −20 °C by a small stainless steel grater with subsequent recovery and mixing 1:5 (*w*/*w*) with a saline solution (1 M NaCl) at 0 °C and subsequent centrifugation at 18,000× *g* for 60 min at 4° C. The oil recovered after centrifugation (supernatant, about 200 µL) was dried over anhydrous sodium sulfate and re-centrifuged at 12,000× *g* for 15 min at 4 °C and stored in the dark in a 1 mL vial under nitrogen at 5° C. A total of 8 samples of essential oils were prepared (all in 2019), 4 of Neapolitan limmo and 4 of lemoncetta Locrese.

### 3.3. Proximate Constituents

The soluble solids, expressed in Brix degrees, were determined by measurement of refractive index at 20 °C. The pH was determined by a Crison Model microTT 2050 pHmeter. Titrable acidity (total acids), expressed as citric acid monohydrate, was determined by titrating a 10 g sample with 0.1 N NaOH up to pH 8.1 according to the method reported by the International Federation of Fruit Juice Producers [46].

### 3.4. Organic Acid Analysis

Limmo or lemoncetta juice (20 g) were clarified by centrifugation at 12,000× *g* for 15 min. The clarified extract was filtered through a 0.45 μm Millipore filter (Darmstadt, Germany); 10 mL was chromatographed through a cation-exchange column [AG-1-X8 (HCOO^−^) poly-prep Bio-Rad (Hercules, CA, USA)] and washed with water to a total volume of 100 mL. The organic acids were eluted with 6 M formic acid (ca. 130 mL), collected, and evaporated. The dry samples were recovered with water (10 mL) and filtered through a 0.45 µm Millipore filter before HPLC analysis. A volume of 10 μL was employed for the HPLC ThermoFinnigan Surveyor (Thermo Finnigan, Waltham, MA, USA) analysis on a Restek Allure organic acids cartridge 5 µm, 300 mm × 4.6 mm ID thermostated at 25 °C. The isocratic elution was carried out with a eluent consisting of 100 mM phosphate buffer at pH 2.5 with a flow rate of 0.5 mL/min with detection at 226 nm by a diode array detector [47] interfaced to a Dell computer Optlex gx260 with Xcalibur software for the signal acquisition and elaboration. Identification of quinic, malic, citric, fumaric, and tartaric acids was based on the retention time by co-injection of reference standards.

### 3.5. Flavonoid Analysis

The determination of the flavonoids (Flavanone *O*-glycosides and Flavone *O*-glycosides) in the juices was carried out by liquid chromatography according to the method of Grandi et al. [27]. Ten phenolic compounds were quantified. They included four flavanone *O*-glycosides with a rutinose (rhamnosyl-α-1,6-glucose) moiety: hesperidin, narirutin, eriocitrin and didymin (or neoponcirin). Four more were flavanone O-glycosides with neohesperidose moiety (rhamnosyl-α-1,2-glucose): naringin, neohesperidin, neoeriocitrin and poncirin. 

Standard solutions of the flavonoids were prepared by weighing exactly 0.1 g of each compound and dissolving it in 100 mL of *N,N*-dimethylformamide. Those solutions were used to build up the calibration lines by diluting them to cover the concentration range of 1–100 mg/L.

The juices (10 mL) were shaken with 20 mL of a 1:1 (*v*/*v*) mixture of 0.25 M *N,N*-dimethylformamide/ammonium oxalate and 20 mL of analytical-grade water and then filtered on 0.45 µm PTFE Pall filters. A volume of 5 µL was employed for the HPLC analysis on a Phenomenex Luna column C18 (l50 × 3 mm) 5 µm thermostated at 25 °C. The elution was conducted as indicated by Grandi et al. [27]. The eluent A was made by an aqueous solution of 5 mM KH_2_PO_4_ adjusted at pH 3.05 with phosphoric acid. The eluent B was obtained by mixing acetonitrile/water/0.25 M KH_2_PO_4_, in the ratio 70:26:4 (*v*/*v*/*v*), and adding 100 µL of H_3_PO_4_ (87%) per liter of solution.

Finally, we quantified two flavone *O*-glycosides with a rutinose sugar moiety: rutin and diosmin. Specific wavelengths were used to identify the individual classes according to Gattuso et al. [48]: flavanones have an absorption maximum at 280–290 nm (set at 285), the flavones rutin and diosmin absorb at 304–350 nm (set at 325).

### 3.6. Chirospecific Analysis of Sabinene, β-Pinene, Limonene, Linalool and Linalyl Acetate in Essential Oils

The analyses were carried out by gas chromatography (GC-FID). The content in sabinene, β-pinene, limonene, linalool, and linalyl acetate of the essential oils of Neapolitan limmo and lemoncetta Locrese was determined by injecting in split modality 1:100 volume of 0.2 µL essential oil diluted 1:10 (*v*/*v*) in acetone. Analyses were conducted with a RTX^®^-5 (Resteck, Bellefonte, PA, USA) column (30 m × 0.25 mm, film 0.25 µm) at 70 °C for 1 min, 3 °C/min at 200 °C, holding for 0.3 min, 15 °C/min at 250 °C, and holding for 5 min. The metabolites content was expressed as the percentage of GC peak areas. Chirospecific analysis of the metabolites was performed with enantioselective multidimensional GC (enantio-MDGC). This technique consists in transferring part of the sample from a primary to a secondary column of different polarity or different chiral type. Our laboratory assembled a MDGC system by joining two Thermo Finnigan Trace 2000 G GC devices through a transfer line thermostated at 160 °C [36]. The first GC was equipped with a non-chiral column, the second had a column with a chiral stationary phase. A six-way rotating valve, positioned in the first GC and switchable via software, diverted the flow coming out the first column either to the first detector (FID 1) or to the second column where the substance enantiomers were separated and monitored with the second detector (FID 2) generating the chiral chromatogram. The non-chiral column employed in the first GC was a RTX^®^-5 column (5% diphenyl/95% dimethyl polysiloxane (30m × 0.25 mm i.d., film thickness 0.25 μm) (Resteck, Bellefonte, PA, USA). The carrier gas was helium at constant flow 1.5 mL/min. Make-up gas was nitrogen at 30 mL/min flow rate. The initial oven temperature was set at 70 °C for 10 min, then it was programmed from 70 to 85° C at 3 °C/min, from 85 to 175 °C at 5 °C/min, from 175 to 285 °C at 6 °C/min and finally at 285 °C for 5 min. The injector temperature was set at 250 °C and the detector temperature (FID) at 280 °C. 

The chiral column used in the second chromatograph was a Diethyl tertbutyl silyl-BETA-Cyclodextrin column (Mega Legnano, Milan, Italy) (25m × 0.20mm i.d., 0.18 µm film thickness). The initial oven temperature was set at 35 °C for 25 min, then it was programmed from 35 to 160 °C at 4 °C/min and held at 140 °C for 2 min. The injector temperature 150 °C and detector (FID) was set at 220 °C. Carrier gas was helium at a programmed pressure. The initial pressure was 290 kpa for 30 min. then varied from 290 to 1500 kpa at 500 kpa/min. For each isomer, the enantiomeric excess (ee%) was calculated as ee% = ((A_max_ – A_min_)/(A_max_ + A_min_)) × 100 where A_max_ and A_min_ are the areas of the more and less abundant isomers respectively.

### 3.7. Volatile Organic Compounds (VOCs)

Determination of VOCs in the juices and peels was carried out by solid-phase micro extraction (SPME) and analyzed with a gas chromatography-mass spectrometry (GC-MS). An automatic injection autosampler CombiPal (CTC-CombiPal Analytics, Zwingen, Switzerland) was used for SPME sampling. The experiments were performed using a 50/30 µm divinylbenzene/carboxen/polydimethylsiloxane fiber (Supelco, Bellefonte, PA, USA). The fiber was conditioned according to the manufacturer’s recommendation to remove contaminants. Before analysis, a fiber blank was run to confirm no contamination peak.

VOCs of juices and peels: An aliquot (8 g) of each juice diluted 20 times was weighed into a 20 mL vial and spiked with 80 µL of internal standard (camphor 3000 mg/L). Each sample was equilibrated at 40 °C for 10 min under stirring (500 rpm). After equilibration, the juice or peel were extracted by exposing the SPME fiber at 40 °C for 10 min (juice) and 2 min (peel). The analytes were desorbed at 250 °C for 15 min in the GC injection port. Measurements were always repeated at least in triplicates.

### 3.8. Gas Chromatography-Mass Spectrometry Analysis

The analyses were performed using a Varian 450 GC (Walnut Creek, CA, USA) coupled with Varian 300-MS mass spectrometer (Walnut Creek, CA, USA). The volatile compounds were separated using a Zebron ZB-semivolatiles capillary column (30 × 0.25 mm i.d., 0.25 µm film thickness). The oven temperature program was set as follows: initial temperature was held at 40 °C for 2 min, increased to 175 °C at 7.5 °C/min, and to 275 °C at 20 °C/min (held for 6 min). The temperatures of the transfer line and the ion source were set at 300 °C and 230 °C, respectively. Helium (99.999% purity) was used as a carrier gas at 0.9 mL/min. A split injection with a ratio of 1:100 was used. The analyses were carried out under full-scan acquisition mode. The mass range used was 30 to 450 *m*/*z*. The identification of volatile compounds was based on the comparison between the mass spectrum for each compound with those of two spectral libraries: NIST (National Institute of Standards and Technology, Gaithersburg, MD, USA) and WILEY (Wiley, New York, NY, USA). The data obtained were collected using the Bruker software Chemical Analysis MS Workstation version 7.0 (Karlsruhe, Germany).

### 3.9. Random Amplified Polymorphic DNA (RAPD-PCR) Analysis

Total genomic DNA of Neapolitan limmo and lemoncetta Locrese was isolated from 10 mg of lyophilized leaves. Plant tissue disruption was achieved at dry ice temperature through stainless beads in 2 mL tubes using a TissueLyser apparatus (Qiagen S.r.l., Milano, Italy) programmed at 30 Hz for 1 min. DNA was extracted according to the GeneJET Plant Genomic DNA Purification Mini Kit (Thermo Fisher Scientific, Carlsbad, CA, USA). The samples were dissolved in pre-heated (60 °C) lysis buffer. The suspensions were incubated at 60 °C for 10 min and subjected to the extraction procedure. RNase A treatment (5 μg/mL) was necessary to eliminate the co-extracted RNA. Finally, the DNA was eluted and diluted a final concentration of 20 ng/μL determined from the UV absorbance at 260 nm. As the purity and quality of the DNA template are crucial factors for a successful PCR, genomic DNA was checked by the 260/280 nm absorbance ratio and agarose gel electrophoresis.

The RAPD-PCR procedure was well established in our group in a previous project on *Citrus*, where sensitivity and reproducibility of the method were examined on genomic DNA from 1 to 100 ng [38,45,49]. In the present study we used 10 ng DNA template samples. The arbitrary primers tested in the PCR reaction had 60% G + C content and were 10 nucleotides long. After an initial screening of 20 arbitrary oligodeoxyribonucleotide decamer primers, 12 primers were selected for reproducibility of the band patterns: A05, AK10, AN10, AX01, AX08, G07, G12, G19, E10, E11, U4, and U19 (Table 6).

Each PCR was carried out in a 50 μL volume, containing 1X DreamTaq buffer with 2 mM MgCl_2_, brought to 3 mM MgCl_2_, 100 μM of each dNTP, 20 pmols of the arbitrary and unique primer, 2.0 Units of DreamTaq DNA polymerase (Thermo Fisher Scientific) and 10 ng of citrus genomic DNA. The PCR mixture was assembled on ice and transferred to a pre-cooled (6 °C) Veriti thermal cycler with a heated lid (Thermo Fisher Scientific).

The DNA template was amplified by the following cycling profile: initial DNA template melting for 3 min at 95 °C, 45 cycles of denaturation for 1 min at 95 °C, primer annealing for 1 min at 40 °C, and synthesis for 1 min at 72 °C. The program ended with a final step conducted for 10 min at 72 °C. The reaction products were stored at −20 °C. Each reaction was repeated three times along with negative controls without genomic DNA. The RAPD-PCR products (25 μL) were separated by electrophoresis on 2% (*w*/*v*) agarose gel containing 0.5 μg/mL SyBr Safe and 1X TAE buffer (89 mM Tris-acetate at pH 8.4, 2 mM EDTA) at 5 V cm-1 for 1.5 h. The GeneRuler 1 kb Plus DNA ladder was used as standard marker of known molecular weights (Thermo Fisher Scientific). Amplicons were visualized under UV transilluminator and digitalized by the Electrophoresis Documentation and Analysis 120 System (Kodak ds-digital science, Rochester, NY, USA).

### 3.10. Statistical Analysis

All samples were analyzed in triplicates and the results were expressed as mean ± standard deviation (SD) after a normality distribution Kolmogorov-Smirnov test. Statistical analyses were performed using SPSS software ver. 21.0 (IBM, Armonk, NY, USA).

Statistical comparisons were carried out by analysis of variance (ANOVA) and post hoc Tukey-Kramer tests. A *p* value less than 0.05 was considered statistically significant. All tests were two tailed.

Significant differences in relative intensities of each volatile compound detected by GC-MS were analyzed by Mann-Whitney U-test between Neapoletan limmo and lemoncetta Locrese (α = 5%).

## 4. Conclusions

In conclusion, chemical and genetic data obtained by DNA fingerprint using RAPD markers have collectively allowed us to confirm beyond doubts that the ancient and rare sweet Mediterranean lime, known in Campania as Neapolitan limmo and in Calabria as lemoncetta Locrese are synonyms of the same citrus species.

Collectively, the obtained compositional data also indicate that, from the chemo-taxonomic point of view, both fruits belong to *Citrus lumia* Risso species despite the di stinct phenotypes. Our results might allow in the future the repopulation of limmo cultures and the reintroduction of this fruit in the essential oil and gastronomic market.

## Figures and Tables

**Table 1:** Flavonoid content (mg/Kg) in Neapolitan limmo and lemoncetta Locrese estimated by HPLC. (n.d. ≤ 0.5 mg/Kg).

Peak	Compounds	Neapolitan Limmo		Lemoncetta Locrese		*p*-Value
		Mean ± sd	Min–Max	Mean ± sd	Min–Max	
1	Eriocitrin (ERC)	166 ± 15	96–229	261 ± 64	176–327	0.003
2	Neo-Eriocitrin (NER)	n.d.		n.d.		
3	Rutin (RUT)	4 ± 1	3–5	9 ± 4	5–12	0.05
4	Narirutin (NRT)	1	trace-2	1	trace-2	
5	Naringin (NRG)	n.d.		n.d.		
6	Hesperidin (HSP)	130 ± 17	45–189	270 ± 80	178–322	0.001
7	Neohesperidin (NHP)	n.d.		n.d.		
8	Diosmin (DSM)	12 ± 3	10–17	24 ± 11	15–30	0.04
9	Poncirin (PON)	n.d.		n.d.		
10	Didimin (DDM)	n.d.		n.d.		

**Table 2:** Proximate constituents. pH, soluble solids (°Brix), titratable acidity (as citric monohydrate acid g/L), and organic acids (g/L) in Neapolitan limmo and lemoncetta Locrese.

Proximate Constituents	Neapolitan Limmo		Lemoncetta Locrese		*p*-Value
	Mean ± sd	Min–Max	Mean ± sd	Min–Max	
Total Soluble Solids	7.9 ± 0.5	7.6–8.5	8.4 ± 0.5	8.0–8.9	0.001
pH	5.8 ± 0.2	5.7–5.9	5.9 ± 0.2	5.8–6.0	0.991
Titratable Acidity	1.22 ± 0.2	0.98–1.32	0.98 ± 0.2	0.84–1.20	0.079
Tartaric Acid	0.15 ± 0.02	0.13–0.17	0.22 ± 0.04	0.17–0.26	0.071
Quinic Acid	0.15 ± 0.05	0.10–0.22	0.32 ± 0.10	0.19–0.43	0.045
Malic Acid	1.57 ± 0.10	1.45–1.70	1.88 ± 0.31	1.42–2.14	0.467
Citric Acid	0.94 ± 0.01	0.85–1.02	0.48 ± 0.25	0.13–0.70	0.003
Fumaric Acid	0.01 ± 0.01	0–0.01	0.01 ± 0.01	0–0.01	0.767

**Table 3:** Enantiomeric distribution of chiral compounds in Neapolitan limmo and lemoncetta Locrese essential oil.

Compound		Enantiomeric Ratio		Enantiomeric Excess, ee (%)	
		Neapolitan Limmo	Lemoncetta Locrese	Neapolitan limmo	Lemoncetta Locrese
β-pinene	*R*-(+)	0.5	0.5	99.2	99.1
β-pinene	*S*-(−)	99.5	99.5		
Sabinene	*R*-(+)	15.4	15.8	69.3	68.4
Sabinene	*S*-(−)	84.6	84.2		
Limonene	*S*-(−)	0.6	0.5	99.2	99.0
Limonene	*R*-(+)	99.4	99.5		
Linalool	*R*-(−)	8.3	83.6	66.9	67.2
Linalool	*S*-(+)	16.7	16.4		
Linalyl acetate	*R*(−)	98.8	98.7	97.6	97.6
Linalyl acetate	*S*-(+)	1.2	1.3		

**Table 4:** Volatile compounds identified in the juices of the Neapolitan limmo and lemoncetta Locrese. Data were expressed as: Area volatile compound/Area Internal Standard (A/As.i.) and Area percent (%). Values correspond to average ± standard deviation. Values are significant at *p* < 0.05.

Peak	Volatile Compounds	Neapolitan Limmo		Lemoncetta Locrese		*p*-Value
		A/A s.i.	%	A/A s.i.	%	
Terpenes						
1	α-phellandrene	0.005 ± 0.00	0.24 ± 0.00	0.007 ± 0.00	0.33 ± 0.00	0.009
2	α-pinene	0.004 ± 0.00	0.18 ± 0.00	0.004 ± 0.00	0.18 ± 0.00	0.06
3	β-phellandrene	0.006 ± 0.00	0.29 ± 0.00	0.009 ± 0.00	0.45 ± 0.00	0.009
4	β-pinene	0.03 ± 0.00	1.38 ± 0.00	0.039 ± 0.01	1.87 ± 0.01	0.009
5	β-myrcene	0.035 ± 0.00	1.62 ± 0.00	0.031 ± 0.01	1.49 ± 0.02	0.009
6	D-limonene	2.051 ± 0.01	93.82 ± 0.01	1.943 ± 0.02	93.39 ± 0.03	0.009
7	β-ocimene	0.002 ± 0.00	0.08 ± 0.00	0.002 ± 0.00	0.10 ± 0.00	0.009
8	Careen	0.001 ± 0.00	0.03 ± 0.00	0.001 ± 0.00	0.04 ± 0.00	0.009
Monoterpenoid Alcohols						
9	Linalool	0.008 ± 0.00	0.35 ± 0.00	0.015 ± 0.00	0.70 ± 0.00	0.009
10	Eucalyptol	0.001 ± 0.00	0.06 ± 0.00	0.002 ± 0.00	0.07 ± 0.00	0.009
11	Isoborneol	0.003 ± 0.00	0.15 ± 0.00	0.004 ± 0.00	0.18 ± 0.00	0.009
12	Borneol	0.001 ± 0.00	0.05 ± 0.00	0.001 ± 0.00	0.06 ± 0.00	0.009
13	Bergamol	0.034 ± 0.01	1.54 ± 0.01	0.015 ± 0.01	0.70 ± 0.01	0.009
Sesquiterpene Hydrocarbons						
14	Caryophyllene	0.001 ± 0.00	0.06 ± 0.00	0.002 ± 0.00	0.08 ± 0.00	0.009
15	trans-α-bergamotene	0.002 ± 0.00	0.09 ± 0.00	0.004 ± 0.00	0.19 ± 0.00	0.009
16	β-bisabolene	0.002 ± 0.00	0.11 ± 0.00	0.004 ± 0.00	0.18 ± 0.00	0.009

**Table 5:** Volatile compounds identified in the peels of Neapolitan limmo and lemoncetta Locrese. Data were expressed as average area of volatile compounds ± standard deviation. Values are significant at *p* < 0.05.

Peak	Volatile Compounds	Neapolitan Limmo (Area)	lemoncetta Locrese (Area)	*p*-Value
**Terpens**				
1	α-phellandrene	5.17 × 10^6^ ± 5.00 × 10^3^	1.42 × 10^7^ ± 5.00 × 10^4^	0.009
2	α-Pinene	3.87 × 10^7^ ± 1.00 × 10^5^	2.82 × 10^8^ ± 2.00 × 10^6^	0.009
3	β-Phellandrene	1.08 × 10^8^ ± 1.00 × 10^6^	7.24 × 10^8^ ± 1.50 × 10^6^	0.009
4	β-Pinene	5.54 × 10^8^ ± 2.50 × 10^6^	4.34 × 10^9^ ± 2.00 × 10^7^	0.009
5	β-Myrcene	4.39 × 10^8^ ± 3.50 × 10^6^	1.66 × 10^9^ ± 5.00 × 10^6^	0.009
6	D-limonene	Off the chart	Off the chart	
7	β-ocimene	4.23 × 10^7^ ± 1.50 × 10^5^	1.27 × 10^8^ ± 5.00 × 10^5^	0.009
8	carene	4.99 × 10^6^ ± 3.50 × 10^4^	8.59 × 10^6^ ± 4.00 × 10^4^	0.009
9	cis-β-terpineol	1.38 × 10^7^ ± 2.50 × 10^5^	2.36 × 10^7^ ± 3.00 × 10^5^	0.009
10	terpinolene	9.53 × 10^6^ ± 1.50 × 10^5^	1.54 × 10^7^ ± 2.00 × 10^5^	0.009
**Monoterpenoid Alcohols**				
11	linalool	6.09 × 10^8^ ± 3.00 × 10^6^	2.47 × 10^9^ ± 2.00 × 10^7^	0.009
12	α-terpineol	1.16 × 10^7^ ± 5.00 × 10^4^	2.05 × 10^7^ ± 1.00 × 10^5^	0.009
13	acetic acid octyl ester	1.31 × 10^7^ ± 5.00 × 10^4^	4.79 × 10^7^ ± 4.00 × 10^5^	0.009
14	trans geraniol	2.28 × 10^7^ ± 3.00 × 10^5^	2.78 × 10^7^ ± 4.00 × 10^5^	0.009
15	bergamol	7.27 × 10^9^ ± 3.00 × 10^7^	1.51 × 10^10^ ± 1.50 × 10^8^	0.009
16	α-terpineol acetate	3.29 × 10^7^ ± 3.00 × 10^5^	3.44 × 10^7^ ± 1.50 × 10^5^	0.009
17	nerol acetate	5.77 × 10^7^ ± 3.00 × 10^5^	5.62 × 10^7^ ± 1.50 × 10^5^	0.009
18	geraniol acetate	7.26 × 10^7^ ± 3.00 × 10^5^	6.22 × 10^7^ ± 5.00 × 10^4^	0.009
**Sesquiterpene Hydrocarbons**				
19	α-bergamotene	7.94 × 10^6^ ± 6.50 × 10^4^	1.12 × 10^7^ ± 1.00 × 10^5^	0.009
20	caryophyllene	3.23 × 10^7^ ± 1.50 × 10^5^	5.56 × 10^7^ ± 2.50 × 10^5^	0.009
21	trans-α-bergamotene	7.44 × 10^7^ ± 2.00 × 10^5^	1.28 × 10^8^ ± 1.00 × 10^6^	0.009
22	cis-α-bisabolene	4.58 × 10^6^ ± 3.50 × 10^4^	6.13 × 10^6^ ± 1.00 × 10^4^	0.009
23	β-bisabolene	5.74 × 10^7^ ± 1.50 × 10^5^	7.71 × 10^7^ ± 1.50 × 10^5^	0.009

**Table 6:** Arbitrary 10-mer primers used for RAPD analysis of Neapolitan limmo and lemoncetta Locrese.

N.	Primer Name	5′-Sequence-3′	GC (%)	Total Bands (nr. 80)	Total Bands (%)
1	A05	AGGGGTCTTG	60	8	10
2	AK10	CAAGCGTCAC	60	6	7.5
3	AN10	CTGTGTGCTC	60	6	7.5
4	AX01	GTGTGCCGTT	60	8	10
5	AX08	AGTATGGCGG	60	12	15
6	G07	GAACCTGCGG	70	4	5
7	G12	CAGCTCACGA	60	9	11.25
8	G19	GTCAGGGCAA	60	8	10
9	E10	CACCAGGTGA	60	5	6.25
10	E11	GAGTCTCAGG	60	5	6.25
11	U4	GACAGACAGG	60	4	5
12	U19	TGGGAACGGT	60	5	6.25
